# Supplementary material for: Comparative Cytogenetic Mapping and Telomere Analysis Provide Evolutionary Predictions for Devil Facial Tumour 2
Source: Genes (Basel). 2020 Apr 28;11(5):480. doi: 10.3390/genes11050480 (PMC7290341; doi:10.3390/genes11050480)
Supplement: Supplementary file 1 [file genes-11-00480-s001.zip › Revised Supplementary figure 1.docx]

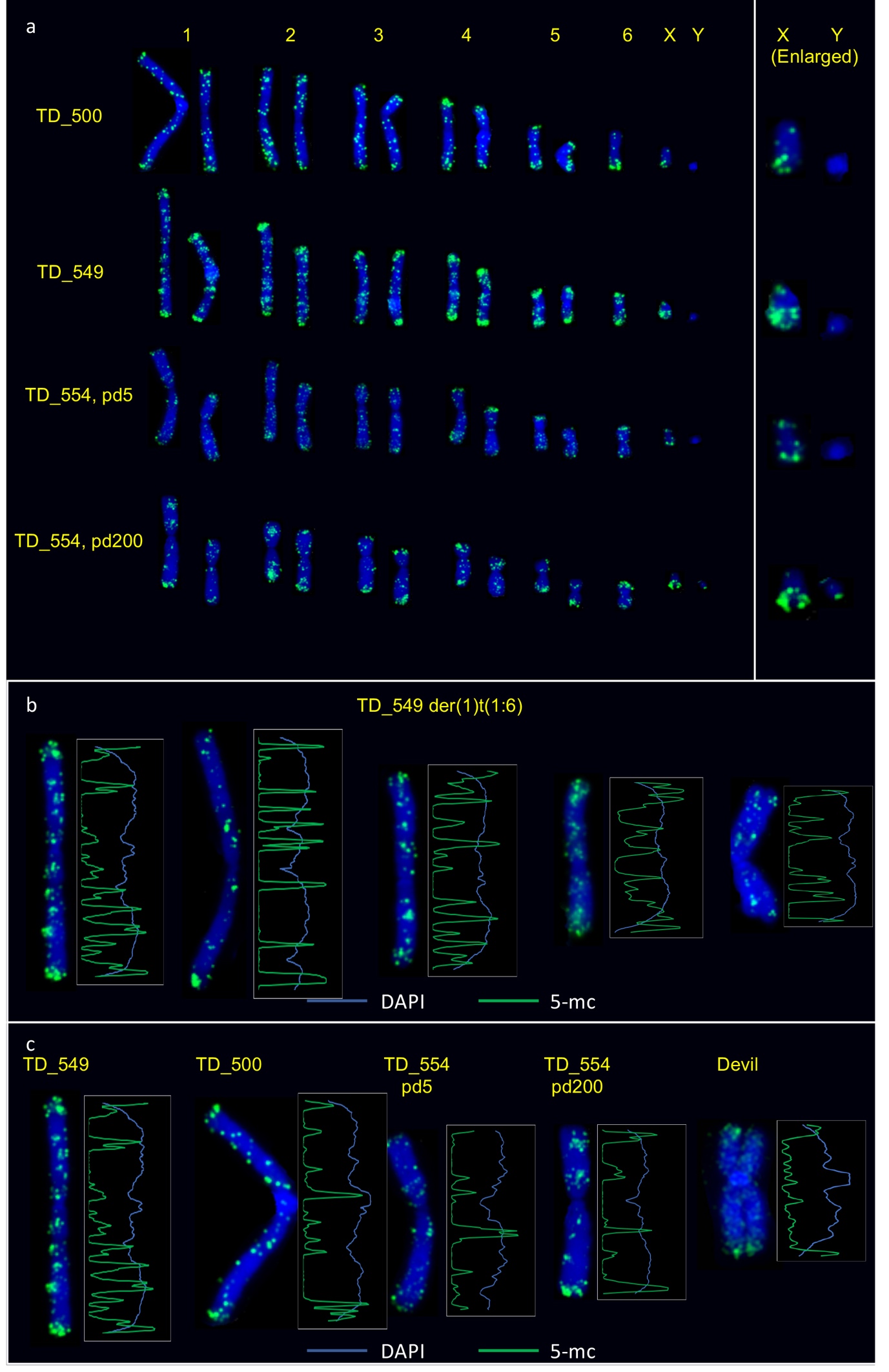


**Supplementary figure 1**. Broad methylation patterns on DFT2 chromosomes. (a) DNA methylation patterns across the karyotypes of three DFT2 tumours (TD_500, TD_549 and TD_554), one after 5 and 200 population doublings (pd 5 and pd 200). TD_500 and TD_549 were analysed after 5 population doublings. Chromosome numbers are denoted along the top row. Anti-5-methylcytosine (5-mC) antibody staining is represented in green and DAPI staining in blue. All tumours exhibit similar staining pattern, with moderate antibody at telomeric ends and weak staining in the chromosome body in all homologues. The X and Y chromosomes are also shown enlarged. Chromosome Y has a noticeable lack of antibody staining, with very little to no signal present. (b) Comparison of anti-5-methylcytosine antibody staining on der(1)t(1:6) from five different metaphase spreads of TD_549 demonstrating the variation in staining. Plot profiler analysis is shown were fluorescent intensity is plotted against the length of the chromosome. (c) Comparisonanti-5-methylcytosine antibody staining patterns on der(1)t(1:6) from each of the cell lines tested (TD_554 at pd5 and pd200) to devil chromosome 1 (Ingles and Deakin, 2015). Visual observation of over 20 metaphases from each tumour line or population doubling indicate there are no consistent changes in broad DNA methylation patterns between any of the tumours, nor population doublings. A sequenced-based approach is needed to more accurately detect and quantify any DNA methylation changes.

Ingles, E.D.; Deakin, J.E. Global DNA methylation patterns on marsupial and devil facial tumour chromosomes. *Molecular cytogenetics* **2015**, *8*, 74, doi:10.1186/s13039-015-0176-x.
