# Supplementary material for: Comparative Cytogenetic Mapping and Telomere Analysis Provide Evolutionary Predictions for Devil Facial Tumour 2
Source: Genes (Basel). 2020 Apr 28;11(5):480. doi: 10.3390/genes11050480 (PMC7290341; doi:10.3390/genes11050480)
Supplement: Supplementary file 1 [file genes-11-00480-s001.zip › DFT2 Supplementary figure 2.docx]

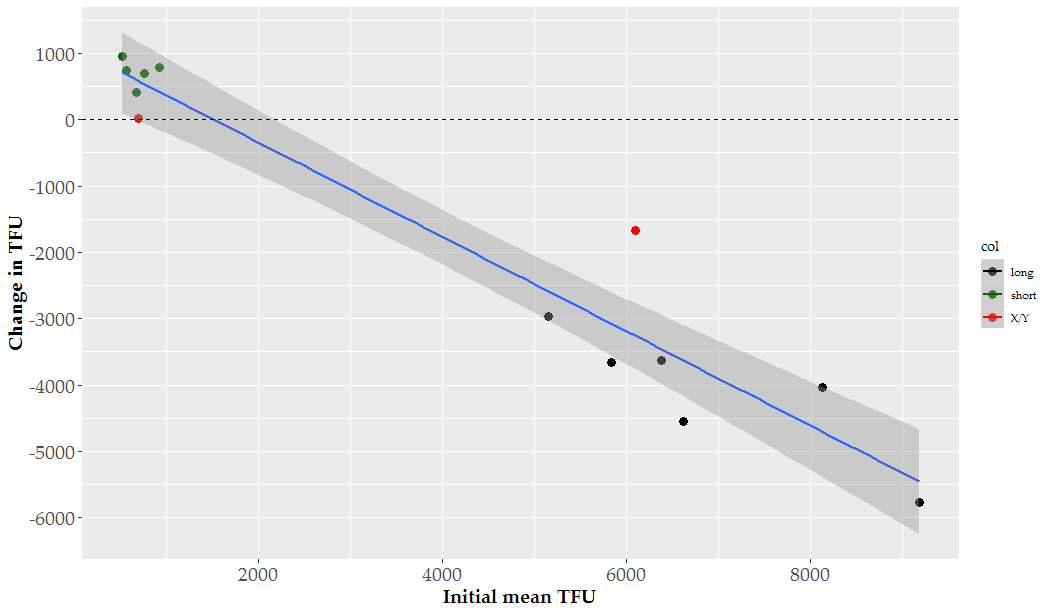


**Figure S2**. Plot of the changes in TFU between population doublings 5 and 200 for each chromosome, divided by length subsets. Changes in the short subset are all small and positive, represented in the cluster at the top left. The long subset have large negative changes in TFU, and form a larger spread out cluster towards the right. Expected change in TFU based on initial mean TFU is plotted as a blue regression line: y = 1078.7338 – 0.7113x (R^2^ = 0.93). Chromosome Y notably deviates from this regression line due to a smaller decrease in TFU.
